# The College of Agriculture and Mechanical Arts

**Published:** 1896-07

**Authors:** 


The College of Agriculture and Mechanical Arts, associated with which at Kingston, R. I., is the State experiment station, provides for its agricultural students a course in veterinary science, but there seems, from the annual report, to be no veterinarian connected with the college faculty or the experiment station. Rhode Island has a number of highly intelligent and well-educated veterinarians, and it would seem right and proper that one of these should be associated with such an important State institution.

